# Proactive Ethical Design for Neuroengineering, Assistive and Rehabilitation Technologies: the Cybathlon Lesson

**DOI:** 10.1186/s12984-017-0325-z

**Published:** 2017-11-14

**Authors:** Marcello Ienca, Reto W. Kressig, Fabrice Jotterand, Bernice Elger

**Affiliations:** 1 Institute for Biomedical Ethics, Faculty of Medicine, University of Basel, Bernoullistrasse 28, -4056 Basel, CH Switzerland; 2 University Center for Medicine of Aging, Felix Platter Hospital, Basel, Switzerland; 3 Chair of Geriatrics, University of Basel, Basel, Switzerland; 4 Center for Bioethics and Medical Humanities, Institute for Health and Society, Medical College of Wisconsin, Madison, USA; 5 University Center for Legal Medicine, University of Geneva, Geneva, Switzerland; 6 Health Ethics & Policy Lab, Department of Health Sciences & Technology, ETH Zürich, Zürich, Switzerland

**Keywords:** Ethics of assistive technology, Proactive ethical design, User-centered, Value sensitive design, Neuroethics, Cybathlon

## Abstract

**Background:**

Rapid advancements in rehabilitation science and the widespread application of engineering techniques are opening the prospect of a new phase of clinical and commercial maturity for Neuroengineering, Assistive and Rehabilitation Technologies (NARTs). As the field enters this new phase, there is an urgent need to address and anticipate the ethical implications associated with novel technological opportunities, clinical solutions, and social applications.

**Main idea:**

In this paper we review possible approaches to the ethics of NART, and propose a framework for ethical design and development, which we call the Proactive Ethical Design (PED) framework.

**Conclusion:**

A viable ethical framework for neuroengineering, assistive and rehabilitation technology should be characterized by the convergence of user-centered and value-sensitive approaches to product design through a proactive mode of ethical evaluation. We propose four basic normative requirements for the realization of this framework: minimization of power imbalances, compliance with biomedical ethics, translationality and social awareness. The aims and values of the CYBATHLON competition provide an operative model of this ethical framework and could drive an ethical shift in neuroengineering and rehabilitation.

## Background

With rapid advancements in rehabilitation science and the widespread application of engineering techniques for the restoration, compensation, assistance and enhancement of human neural systems, the field of neuroengineering is entering a new phase of clinical and commercial maturity. The first pioneering research prototypes of the 1980s and 90s have evolved into an increasingly mature technological spectrum with direct clinical applications and corroborated efficacy. Over the past two decades, assistive and rehabilitation technologies have increased in number and variety. Concurrently, many invasive and non-invasive neurotechnologies have become available for assistive and rehabilitation aims. This expanded technological domain might be regarded as Neuroengineering, Assistive and Rehabilitation Technology (NART). NARTs have been developed with the main purpose of mitigating several morbidities associated with diseases and traumatic injuries to the human nervous system. Today, this evolving spectrum encompasses five major technological families: devices for robot-assisted training, functional electrical stimulation (FES) techniques, prosthetics, brain-computer interfaces (BCIs) and powered mobility aids, many of which were listed as competing disciplines in the CYBATHLON 2016 [1].

Many of these applications have shown efficacy in improving neurological care and neurorehabilitation in relation to a number of functional domains. For example, randomized controlled trials performed on robotic devices for post-stroke therapy and rehabilitation showed that NARTs can enable significant improvements in the therapeutic outcomes compared to usual care [2], especially with respect to motor function [3] and quality of life [4]. In parallel, at the commercial level, several neuroengineering tools for assistance and neurorehabilitation have made their way onto the market and are now available as effective tools for neurological care and rehabilitation. The InMotion ARM™ robot, for instance, allows the efficient delivery of personalized intensive sensorimotor therapy to neurologic patients who need upper-limb rehabilitation while the Lokomat® powered robotic gait trainer has shown effectiveness in improving locomotor gait-training for patients with incomplete spinal cord injury.

As the field of NART enters a new phase of clinical and commercial maturity, many authors have urged to address the ethical implications of this emerging field.

In a recent report based on the outcomes of a joint workshop between the US National Science Foundation and the German Research Foundation on “New Perspectives in Neuroengineering and Neurotechnology”, a group of international experts identified key technological, social and ethical challenges to the adoption of NARTs in the clinical setting. They concluded that the envisaged progress in neuroengineering requires a careful reflection on the ethical and social implications, in particular in relation to issues such as safety, security, privacy, public acceptance and respect for autonomy [5]. In a similar fashion, participants of an interdisciplinary symposium at the NeuroTechnology Center (NTC) at Columbia University have advocated for the integration of ethics into neurotechnology and recommended the development of ethical guidelines for developers and users of novel products [6]. This need for ethical guidelines has not been advocated only by researchers and scientists but also by rehabilitation professionals. Nijboer et al. have investigated the views of rehabilitation professionals and other stakeholders on the use of BCIs (one of the six disciplines featured in the CYBATHLON 2016) as assistive technologies. Their findings show that professionals are urging developers to carefully consider ethical and socio-cultural issues at the level of design [7]. In addition, the lack of ethical consideration is increasingly seen as a major barrier for technology transfer of BCIs as assistive technology in neurorehabilitation [8].

Although it has only recently become an object of empirical and normative investigation, the need for ethical analysis in clinical neuroengineering is not a new demand but one that is deeply rooted in the neurorehabilitation practice. In fact, ethical significance is inherent to the very objectives and mission of the neuroengineering enterprise. As the goal of clinical neuroengineering and neurorehabilitation is to restore, repair, assist and enhance the capabilities of people with neurological conditions, its very mission is of primary ethical relevance and implicitly incorporates moral principles such as promoting end-user’s autonomy, wellbeing and independence, empowering them across a wide range of activities and reducing their social isolation. This predominantly beneficence-oriented and autonomy-oriented ethical goal is well captured by the mission of the Rehabilitation Engineering and Assistive Technology Society of North America (RESNA). RESNA’s mission statement, in fact, emphasizes the aim of improving the potential of people with disabilities to achieve their goals through the use of technology.^1^ An ethics-laden language is also at core of the Cone Health Neurorehabilitation Center, where a stroke support group was recently established for newly diagnosed patients “to make certain they feel *empowered* to take charge of their health and wellness to live a *full life*”.^2^

In addition, the clinical implementation of NART raises ethical attention because the end-user population of these technologies is largely composed by vulnerable individuals with neurological conditions and other functional variabilities that, in virtue of their vulnerability, are often entitled to extraordinary ethical protection. For example, clinical BCIs can be used by individuals with advanced neuromuscular disorders, including patients with locked-in syndrome [9], while robot-aided rehabilitation provides effective support during the recovery process of patients following a stroke [10].

Finally, as the pace of development of new technological products is reportedly faster than their social adoption and ethico-legal assessment, there is a risk that the beneficial potential of NART remains under-expressed if social, ethical and legal implications remain unaddressed. This is particularly relevant for potentially disruptive sociotechnological trends such as assistive robotics as well as for technologies ─such as invasive BCIs─ that establish direct connection pathways with the human brain, hence raising delicate ethical questions about integrity, mental privacy and personhood [11]. A recent review about responsibility in rehabilitation robotics (including neurorehabilitation robots, robotic prostheses, and even next-generation personal assistance robots), has observed that most devices operate in close proximity or direct physical contact with patients, manipulate instruments inside their bodies or directly move their impaired limbs, and have invasive or non-invasive connections with the human nervous system [12]. This raises the need for high ethical attention. While there is an increasing consensus among scientists, engineers and clinicians that ethics is relevant for NART, several conceptual and practical obstacles prevent the successful incorporation of ethical factors into product design and development.

First, at the conceptual level, it is often unclear what ethical considerations should be prioritized and at what level of the technology development process (e.g. design, clinical trials, or post-commercialization assessment).

Second, at the practical level, ethical guidelines and ethics-oriented clinical recommendations remain rare. For example, the RESNA Strategic Plan 2014–2018 does not address ethical considerations and even the RESNA Code of Ethics provides only eight general integrity guidelines to guide the conduct of members and service providers but remains silent on how to incorporate ethics into technology or how to maximize ethical values through their applications.^3^ Similarly, the IEEE Engineering in Medicine and Biology Society (EMBS), the world’s largest international society of biomedical engineers, provides a set of rules for ethical conduct in research but does not address substantive ethical considerations associated with technology use. In other words, existing guidelines often focus on how to ethically develop assistive technologies. However, little guidance is available to engineers and researchers on how to develop ethical assistive technologies, that is technologies that promote ethical values.

Third, in many assistive domains such as the support and rehabilitation of elderly adults with physical or cognitive disabilities, ethical design remains reportedly sporadic [13] while ethical assessment and compliance with guidelines are often perceived by developers and manufacturers as delay factors in the process of development and commercialization of new products.

In this paper we review possible approaches to the ethics of NART and propose a framework for ethical design and development, which we call the Proactive Ethical Design (PED) framework. We also suggest that the aims and values of the CYBATHLON [1] provide an ostensive and operative model of this ethical framework.

It is important to highlight that the ethical challenges raised by assistive and rehabilitation technology are not necessarily unique but might apply also to other sectors of medical technology. Nonetheless, the repeated calls for ethical guidelines advocated by experts’ committees and the relative infrequency of ethical guidelines in professional codes indicate a need for a proactive and collaborative framework that could facilitate the successful design, development and implementation of assistive and rehabilitation technology in an ethically responsible manner.

## Reactive vs. proactive ethics of assistive technology

The ethical aspects of NART can be approached either reactively or proactively. Reactive approaches focus on the critical ethical evaluations of novel products and the assessment of their compatibility with existing normative ethical principles. In reactive ethics, ethical conflicts or problems are addressed as they arise, which usually occurs only at the end of the development process when the finished system is being implemented. For example, authors have performed ethical assessment of commercially available consumer-grade BCIs and argued that their security vulnerabilities may conflict with the principle of informational privacy [11, 14].

In contrast, proactive approaches are characterized by the development of strategies and solutions before a new technology becomes a source of potential ethical confrontation or conflict. Instead of merely reacting to an existing ethical problem, proactive approaches anticipate future potential uses, requirements, and unintended consequences of new technologies before they become ethical issues. For example, Bonaci et al. (2015) have anticipated an operative solution to the privacy vulnerability of commercial BCIs and developed a system called *BCI Anonymizer* that integrates privacy safeguards into the BCI headset [15], hence proactively promoting the ethical principle of respect for privacy.

The notion of proactive ethics was independently coined in the fields of, respectively, business ethics and clinical ethics consultation. In business ethics, the notion “proactive” is used when a business introduces ethical measures (e.g. transparency, accountability and communication) before the eruption of crisis situations, rather than in response to the crisis [16]. Similarly, in clinical ethics consultation, this notion is used to describe a process-oriented approach to ethics consultation (e.g. in ICUs) where communication and planning begin prior to crises [17]. Pavlish et al. (2013) have further developed this notion into a Proactive Ethics Framework, that is a comprehensive set of proactive, ethics-specific, and evidence-based strategies for mitigating ethical conflicts in the clinical setting [18]. This framework included sequential key action points, beginning with the creation of an ethics-minded culture, and continuing with the implementation of risk reduction strategies and the response to early indicators.

Reactive and proactive approaches are not necessarily mutually exclusive but can be complementary. As the example above shows, they can be two sequential phases of a continuing technology assessment process: first, in the reactive phase, ethical conflicts are identified and assessed; concurrently, in the proactive phase, further ethical considerations are anticipated and ethically relevant solutions are incorporated into the design of novel products.

The advantage of reactive approaches to the ethics of neuroengineering is that they allow ethicists and engineers to optimize their efforts and focus on concrete problems rather than on the anticipation of possible future scenarios that are often hard to foresee. However, reactive approaches ─if not in conjunction with concurrent proactive considerations─ present several disadvantages. First, they are structurally postdated since they provide ethical advice, by definition, only at the post-development level [19], that is at a stage when there is little or no room for modification of a NART device. Second, in several domains of cognitive and physical disability such as dementia and age-dependent frailty, the lack of proactive ethical and social considerations has been inferred as a determinant of low adoption and acceptance of technology [20, 21]. In fact, if the impact of ethically relevant factors is not anticipated, products might not match the end-users’ needs and wishes, hence result in sub-optimal uptake, implementation lag and delayed clinical or social benefit. Third, there is a risk that lack of proactive ethical considerations may cause negative public perceptions or even unjustified Luddite fears among end-users, caregivers and other relevant stakeholders [22]. This risk is particularly concrete in relation to advanced technologies such as those that incorporate or embed Artificial Intelligence, as their underlying mechanisms and functionalities are often unclear to users [23]. Finally, reactive approaches are a possible source of antagonism and conflict between designers and developers, on the one hand, and ethicists and policy makers, on the other hand. The reason for that stems from the fact that, in a reactive context, engineers and ethicists may engage in a competitive dynamic where the work of the former professionals is being constantly questioned and judged by the latter. By contrast, in a proactive approach, all parties are encouraged to work together. It is worth considering, however, that even though proactive approaches encourage interaction among ethicists and engineers, they are not *necessarily* conductive to collaborative approaches.

## Modes of proactive ethics: User-centered and value-sensitive design

In most circumstances, the type of approach to the ethics of NART chosen by manufacturers is influenced by the process of product design. For example, the increasing prevalence of bottom-up and user-driven approaches to the design of NARTs has been often observed to “move a step further to the ethics of the user” [24], reduce usability problems or conflicts ─since these can be identified and resolved before the systems are launched─ and facilitate the incorporation of ethical considerations in the design process [13]. This suggests that the type of technological design adopted by manufacturers is not morally neutral but determines the possibilities of an assistive technology and has consequences for human wellbeing [19].

**Fig. 1:**
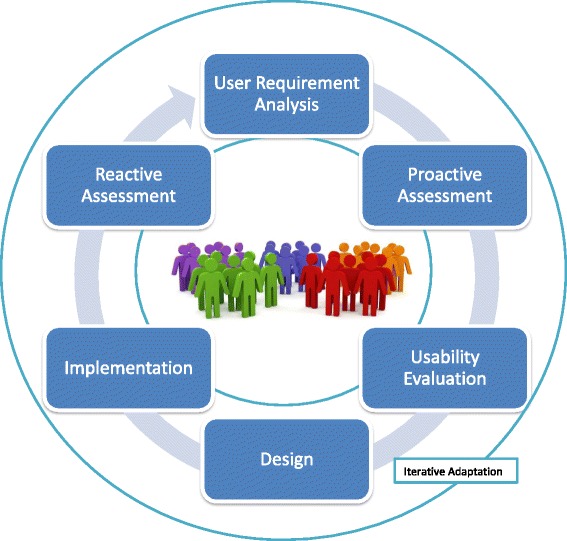
A Visual Representation of the Iterative Dynamics of User-Centered Design

The “user-centered” (sometimes also referred to as “patient-centered”) approach is a framework of processes for the design and development of assistive technologies in which the needs, wishes, and limitations of end-users are given extensive attention at each stage of the design process [25] (Fig. 1). The user-centered (UC) family encompasses a number of methodologically contiguous approaches including cooperative design (where designers and users are involved on an equal footing), participatory design (where users are involved through active and participative processes) and contextual design (where the participatory process occurs in the actual context or environment). For example, the Us’em wearable device, a rehabilitation tool for motivating stroke patients to use their impaired arm-hand in daily life activities, was designed and developed using an user-centered process during which stroke patients, therapists, rehabilitation researchers, and interaction design experts were actively involved [26].

UC approaches are being increasingly considered a necessary requirement for ethical design of NART [8, 27]. The reason for that is twofold.

First, by putting users at the center of design and development, UC approaches shift the location of power in the research process [28]. Through this approach, users are no longer conceptualized as passive recipients of a new product who are implicitly coerced to change their behavior to accommodate the new technology. In contrast, they are empowered at each stage of the design and development process (requirement analysis, pre-production models, mid-production and post-production). In addition, they are no longer subordinated to designers in the decision-making process regarding a new technology, but actively involved in a cooperative dynamic and on a potentially equal footing.

Second, at the practical level, UC approaches facilitate the translation of new assistive technologies into standard rehabilitation practice and care, hence accelerate and maximize the social and clinical benefits of technological innovation. In fact, the translation of new technologies from the designing lab to the rehabilitation clinic can best be accomplished if a patient-centered focus is incorporated throughout the research and development continuum and changes are made so that biomedical innovation serves the broadest needs within the shortest period of time [29]. This societal outcome is consistent with multiple ethical principles and theories. For example, it is consistent with Stuart Mill’s principle of aggregate utility, the foundational ethical tenet of classic utilitarianism, according to which people desire happiness —the utilitarian end— and where general happiness is considered “a good to the aggregate of all persons” [30].

Third, in determining this shift in the location of power, UC approaches inherently promote ethical principles, especially the respect for autonomy, which is one of the four fundamental principles of biomedical ethics [31]. At least two components of personal autonomy are promoted through UC design: decisional autonomy and executional autonomy [32]. Decisional autonomy is the capability to make decisions without restraint from other actors or pre-imposed designs. This capability is promoted if users are actively involved in the decisional process of product design and enabled to make choices or suggestions based on their wishes and needs. Executional autonomy is the capability to act according to a desired course of action. This capability is promoted if users can successfully use assistive technologies tailored around their needs and wishes, hence become able to perform tasks that they might not be able to perform otherwise.

However, authors have argued that decisional and executional autonomy might not be sufficient to guarantee full autonomy and participation of users in rehabilitation. Rather, another component of the autonomy concept is required, that is self-realization [32]. According to this notion, users should not only be granted the capability to make free decisions and act independently, but should also be able to shape their life “into a meaningful existence which expresses individuality” (p. 972). Patients who need NARTs may be experiencing a reduction in their capacity to act as autonomous persons along all these three dimensions (decisional, executional and self-realization). Therefore, NARTs may compensate for such reduced capacity and boost patient autonomy.

Considerations of this kind have led researchers to complement the user-centered framework with values of psychological and ethical significance. The resulting systematic approach is called value-sensitive design (VSD) and is characterized by the embedment of human values into technology design. In the VSD approach values are defined as the “principles or standards of a person or society, the personal or societal judgment of what is valuable and important in life” [33].

According to the VSD approach, NARTs should embody and account for ethical, social and psychological values “through a theoretically grounded approach in a principled and comprehensive manner throughout the design process” [34]. VSD has often been described by engineers, clinicians and ethicists as a successful strategy to incorporate ethics in the overall design process of assistive and rehabilitation technology [19, 35]. In light of this, VSD approaches have raised increasing interest among researchers, a phenomenon confirmed by a fivefold increase in research papers in the field of human-computer interaction mentioning “human values” during the past ten years [36].

Recently, ethicists of healthcare technology have tried to operationalize the principles of VSD in the context of assistive and rehabilitation technology. For example, van Wynsberghe has used the blueprint of VSD “as a means for creating a framework tailored to care contexts”. These efforts are motivated by the need of guaranteeing that NARTs enter the clinical domain in a manner that “supports and promotes fundamental values” in healthcare [35].

While having the merit of enhancing the ethical sensitivity of emerging assistive technology, neither the UC nor the VSD approach are anchored by default on a specific normative grounding or ethical theory [37]. Rather, they can be realized through multiple normative principles or ethical theories. In addition, it has been observed that differences exist between designers’ values and users’ values [38]. This raises the question of how to implement VSD approaches in a multi-cultural society where people could reasonably disagree on important values. While we recognize the importance of the problem, in this paper we refer to VSD as a method “that can be applied in principle to any set of values” and not as the “methodological instantiation of a particular set of values” [36]. Future ethical research should discuss which ethical values (e.g. universal vs. culturally-relative) should actually be instantiated in NARTs.

While we remain agnostic about the specific instantiation of ethical values in the strong sense, in the following, we propose a UC and VSD approach to ethical assistive and rehabilitation technology based on four basic normative requirements. We call this approach the Proactive Ethical Design (PED) framework. Finally, we refer to the experience of the CYBATHLON 2016 competition as an ostensive and operative model of this ethical framework.

## A framework for proactive ethical design

There is an increasing consensus that UC and VSD are necessary requirements for ethically sustainable development of assistive and rehabilitation technology [7, 8, 13]. However, little analysis is available on the prerequisites of successful adoption of such approaches. Based on the inherent goals and objectives of UC and VSD described above, we argue that four basic normative requirements are necessary for the successful implementation of ethical NART.

### Minimization of power imbalances

Both UC and VSD presuppose the minimization of power imbalances in decision-making and a certain degree of inclusiveness and democratization in the design process. This shift in the location of power across the technology design continuum is best achieved through a goal-oriented cooperation among designers, developers and end-users. This principle implies that in order to be involved on an equal footing in the design process, all stakeholders should be incentivized to share common goals that could be pursued through coordinated and cooperative efforts. In fact, in absence of common goals or even in presence of mutually conflicting objectives between different stakeholders (e.g. designers vs users), no successful cooperation within the UC and VSD framework is likely to occur. An example of conflicting objectives between different stakeholders is the observation that designers and developers often prioritize the effectiveness of a new technology whereas users often prioritize usability. Effectiveness refers to the accuracy and completeness with which end-users can achieve certain goals in a certain environment. Usability is the easiness and extent to which a technology can be used by users to effectively achieve these goals. This discrepancy between effectiveness and usability has been particularly investigated in the context of assistive BCI, one of the technologies featured in the CYBATHLON 2016 [1]. For example, a review of BCIs as access pathways for people with severe disabilities has shown that most current prototypes are developed with focus on speed and accuracy instead of usability [39]. These conflicts of objectives can have detrimental consequences for rehabilitation as they could concur in the phenomenon of technology abandonment. This refers to the fact that users of an available assistive or rehabilitation technology might stop using it after an initial phase, a phenomenon that is particularly common with technologies for home use. Scherer has reported that about one third of all assistive technologies are abandoned, and many others might continue to be used sub-optimally due to unease and discomfort. As she states: “we have no information about the number of people who continue to use devices they are unhappy or uncomfortable with because they cannot abandon them without facing more severe consequences” [40]. In addition, the absence of common objectives among different stakeholders involved in the design and development of assistive and rehabilitation technologies is likely to cause the so-called “problem of many hands” [41]. This problem denotes the risk that in complex process where multiple stakeholders are actively involved errors can be made although no class of stakeholders acted in an explicitly reckless or negligent way.

To overcome this problem, there is a need for harmonizing the objectives of all relevant stakeholders involved in the design process through an iterative and dialogic confrontation. This could be achieved by creating cooperative scenarios where all stakeholders are incentivized to pursue a common goal or objective.

### Compliance with biomedical ethics

The second requirement for the successful implementation of ethical assistive technology in rehabilitation is compliance and coherence with biomedical ethics. NARTs are integral part of biomedicine and biotechnology. Nonetheless, their degree of ethical scrutiny by biomedical ethicists is often lower compared to other domains of biomedicine and biotechnology such as pharmacological interventions. This is probably due to many factors including the relative novelty of NART, a less stratified history of misuse and different risk-related perceptions among professionals.

We argue that successful technology development via UC and VSD presupposes the compliance with biomedical ethics. As we said before, this requirement can be fulfilled through compliance with multiple approaches and values in biomedical ethics such as utilitarianism, Kantianism or virtue ethics. Among others, one viable and, according to some, easy-to-implement approach is principlism, a practical approach for ethical decision-making that focuses on four common-ground moral principles: beneficence, non-maleficence, autonomy and justice. Research shows that the principlist approach has the largest circulation among health professionals and the highest prevalence in ethics curricula for health science students [42, 43]. This fact could, ceteris paribus, guarantee better acceptance and easier implementation among health professionals. However, it is important to highlight that, at any rate, referring to any specific ethical theory in a predetermined manner risks to preempt normative input from users. Therefore, it is important that, at any rate, ethical theories or principles are chosen based on the needs and values of users, and adapted to these needs and values through an iterative and flexible process. In other words, the investigation of the users’ needs and values should determine which ethical content is most suitable for a certain technology in a certain patient population, not vice versa.

Principlism, uses a “common morality” approach and “mid-level” prima facie principles: beneficence, non-maleficence, respect for autonomy and justice [31]. Beneficence is the promotion of the wellbeing of people with disability through the successful implementation of assistive and rehabilitation technology. As we have seen above, the field of assistive and rehabilitation technology urges a broad concept of beneficence that is not only focused on the effectiveness of new technologies but also on their usability.

Non-maleficence is the principle of preventing or minimizing harms associated with the use of assistive and rehabilitation technology. This principle is promoted through the implementation of safeguards for the safe and secure use such as the precautionary approach, namely the idea that technologies whose consequences are difficult to predict should be first investigated in a safe setting [19]. Neurorehabilitation experts have tried to systematize the principle of non-maleficence in relation to robot-assisted neurorehabilitation [44]. Their model is based on the postulation of three fundamental laws called the laws of neurorobotics in rehabilitation, a re-elaboration of Asimov’s laws of robotics [45]:

(I) A robot for neurorehabilitation may not injure a patient or allow a patient to come to harm.
(II) A robot must obey the orders given it by therapists, except where such orders would conflict with the First Law.
(III) A robot must adapt its behavior to patients’ abilities in a transparent manner as long as this does not conflict with the First or Second Law.

The first law postulates that rehabilitation robotics should be safe not only in terms of movement, but also from other medical points of view. This can be achieved by designing new products in accordance with international standards such as ISO 13482:2014 [46] and through careful consideration of unintended harms, where harm is understood as any “possible damage to patients” including discomfort and time spent on ineffective rehabilitation. The second law postulates that assistive technologies should not replace therapists, but rather complement existing treatment options. Therapists should always be on the loop of robot-assisted rehabilitation and maintain a position of control in relation to the adjustment of technological parameters, the avoidance of harmful compensation strategies and identification of trade-offs between rehabilitative goals and the psychological dimension of patients. Risks of reduced control over technological parameters such as is the discrepancy between the desired and actual values of some parameters of the electromechanical Gait Trainer [47] should be prevented. At the same time, based on the third law, automatic features and artificial intelligence might be used to support rehabilitation therapists by performing all the control changes required for a successful therapy.

The principle of respect for personal autonomy, as stated above, should not be seen exclusively as the promotion of decisional and executional autonomy, but of self-realization as well. To achieve that, UC approaches should not only involve the active participation of end-users and investigate their perceptions only in relation to quantitative parameters such as effectiveness and usability, but should proactively incorporate user-driven ethical and psychological factors in product design. Given the requirements of context-sensitive design, this attempt to “materializing morality” [48] through assistive technology should be dependent on the specific context and environment of end-users.

Finally, justice is the principle of biomedical ethics that requires assistive technologies to be fairly accessible to users, affordable across various socioeconomic classes, and evenly distributed across rehabilitation clinics in various world regions. While this principle can be incorporated into product design by favoring scalable, low-cost and pervasive technologies, yet design alone might be insufficient. In addition to that, justice-promoting policies should be pursued at various levels of health-technology regulation. Reimbursement policies and State incentives have been advocated elsewhere as possible justice-promoting regulatory interventions [27].

### Translationality

The third requirement is translationality. In fact, the ethical goal of maximizing wellbeing for all individuals with disability through the use of NART is highly dependent on the process of translating research from the designing lab to the rehabilitation center. In order to maximize the societal benefits of NART, we need to ensure that new technologies actually reach the patients or population for whom they are intended and are implemented correctly [49]. Slow or incomplete translation across bench, bedside and community ─ which the European Society for Translational Medicine calls the “three main pillars” ─ is likely to reduce the beneficial impact of assistive technology on the global healthcare system. According to the Institute of Medicine’s Clinical Research Roundtable, two distinct phases in the translational process are in particular need of improvement: the first translational block (T1) prevents basic research findings from being tested in a clinical setting; the second translational block (T2) prevents proven interventions from becoming standard practice.^4^

### Social awareness

Finally, the fourth requirement is raising social awareness and favoring knowledge dissemination across society. The public is often skeptical or reluctant regarding the use of new technologies because of lacking knowledge on the technology and its applications [50]. Sociologists have identified historical patterns and dynamics of opposition to technological innovation. For example, Juma has explored the multi-layered dimensions of socio-political resistance to various types of technological innovation including biomedical technology. These include established social norms, financial considerations, health implications, social disruption, as well as prejudices or human ignorance [50]. Patterns of resistance to new technologies have also been observed in the specific context of healthcare technology [51]. This opposition seems to be particularly significant in relation to technologies that operate in proximity to the human body such as wearable devices and neural prosthetics. A 2014 Pew survey showed that 53% of Americans think it would be a bad thing if “most people wear implants or other devices that constantly show them information about the world around them.” In contrast, just over one third (37%) think this would be “a change for the better” [52]. Since many NARTs operate in close proximity or direct physical contact with patients, and have invasive or non-invasive connections with the human nervous system, they are likely to be affected by these negative public perceptions.

**Fig. 2:**
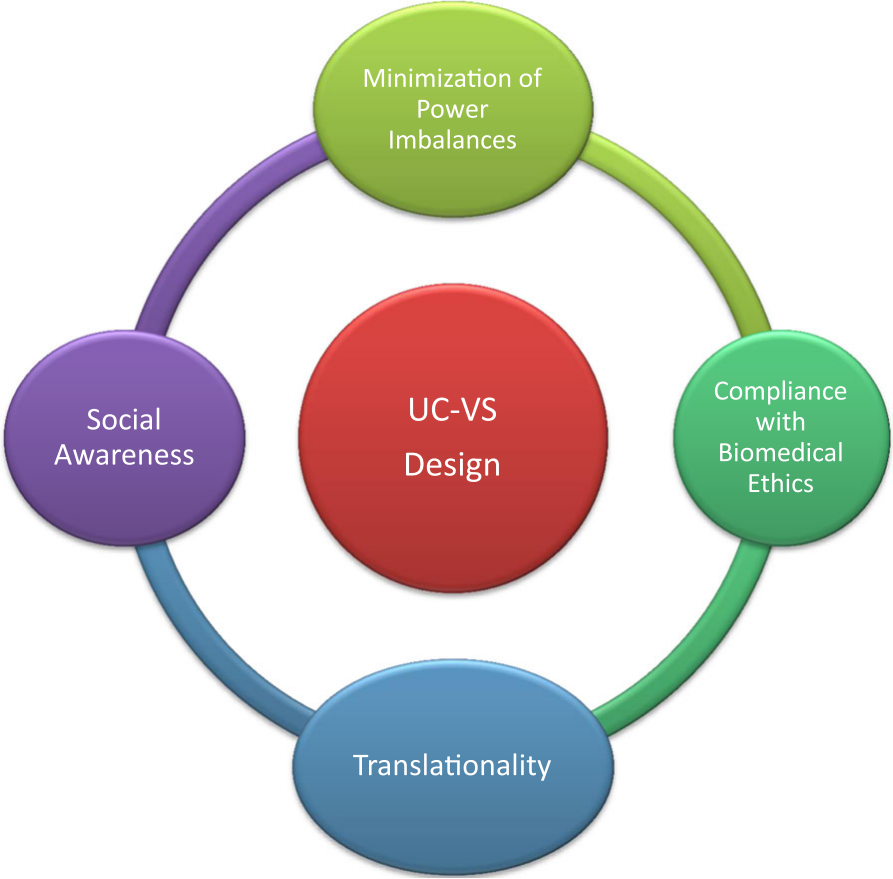
A Framework for the Proactive Ethical Design (PED) of Neuroengineering, Assistive & Rehabilitation Technology

The media, a major catalyzer of attention and knowledge on novel technological possibilities, have started only recently to properly cover the domain of neuroengineering, assistive and rehabilitation technology. Concurrently, since NARTs are still in an initial phase of the technology life cycle, their pervasive implementation might still be limited by enduring habits of health professionals, financial limitations and issues of resource allocation or conservative managerial decisions ─all phenomena that have already been observed in other sectors of healthcare technology [53–55]. If improving the effectiveness, usability and ethical potential of assistive technology is the grand challenge for neuroengineering, raising social awareness is the corresponding societal challenge. It is worth stressing that these requirements should not be seen as values per se, but as *conditions of possibility* for the consideration and incorporation of values through UC and VSD (see Fig. 2). In fact, we hypothesize that UC and VSD approaches cannot be properly implemented if: (i) major power imbalances persist, (ii) biomedical ethics is ignored, (iii) prototypes are not adequately translated into viable products for users and (iv) there is a lack of social awareness about the clinical benefits. However, we recognize that this causal relationship can be bidirectional as: (i) the four normative requirements enable UC & VSD, but, in parallel, (ii) the adequate realization of UC & VSD guarantees the fulfillment of the four normative requirements.

## Proactive ethical design: The Cybathlon lesson

In October 2016, ETH Zurich organized in Zurich, Switzerland, the first edition of the CYBATHLON, an international championship for competitors with disabilities using bionic assistive technologies. The competition featured six disciplines – a Functional Electrical Stimulation (FES) bicycle race, a Powered Leg Prosthesis Race, a Powered Wheelchair Race, a Powered Exoskeleton Race, a Powered Arm Prosthesis Race, and a BCI neurogaming race [1].

We argue that this innovative event represents an ostensive and operative model of the ethical framework delineated in this paper. The reason for that stems from the fact that the CYBATHLON embodies all four required approaches for the successful implementation of ethical NART in rehabilitation.

First, the CYBATHLON model fulfills the first requirement by providing an ideal setting for a goal-oriented cooperation among different stakeholders. During the CYBATHLON 2016 competition, designers, developers and end-users have not only engaged in cooperative dynamics on an equal footing (as required by the UC approach) but also shared a common goal. This created a goal-converging dynamic where the success in the race of the user (the competing athlete) corresponds to the success of the designing team. Such gamification creates a fruitful and possibly reproducible setting for harmonizing the objectives of all relevant stakeholders involved in the design process. Concurrently, it shifts the location of power by putting the user (the individual athlete with disability) at the center of the arena. This centrality of the user in the competition is an ultimate form of empowerment: instead of being a passive recipient of technology-assisted rehabilitation, the person with disability becomes the protagonist of a cooperative process.

Second, the CYBATHLON model fulfills the second requirement by proactively anticipating compliance and coherence with the principles of biomedical ethics. The day prior to the competition, a roundtable discussion involving end-users, patient and industry representatives also hosted a prominent ethics researcher. In addition, the creation of a goal-oriented cooperation between designers and athletes facilitates the promotion of beneficence, non-maleficence and patient autonomy by giving them the possibility to request adaptations of the prototypes according to their wishes and needs at every stage of the process. This iterative process of needs assessment and product adjustment exemplifies the ideal feedback-loop between designers and users that should be pursued in the research setting according to the UC and VSD frameworks. While beneficence is captured by the need of increasing efficiency, effectiveness and usability in order to win the competition, and the non-maleficence principle is embodied by safety-enhancing safeguards, the autonomy of users is maximized by their physical and decisional centrality in the process. As a factor of limitation, the justice principle occurred more sporadically during the CYBATHLON 2016 due to multiple facts: (i) high-performing technologies are likely to be financially expensive; (ii) the competition took place in one of the world’s wealthiest countries; (iii) most competing teams were from affluent and highly industrialized nations. Future editions of the competition should compensate for this omission and incorporate the justice principle, for example by creating a component of the competition involving low-cost technologies, hosting the event in non-European and non-North American countries and encouraging participation of research teams from emergent and developing countries.

Third, the CYBATHLON competition fulfills the translationality requirement by enabling a smooth and accelerated translation of innovative research in assistive technology for the benefit of individual users and the community. Each competing team in the CYBATHLON championship is a small-scale translational round-block that translates research findings into utilizable technology and assesses them in a public arena together with real end-users. This translational power is corroborated by the possibility that through the CYBATHLON competition many technologies originally designed for a small-sized group of people with disability may found an application in larger markets including people with similar functional disabilities or even able-bodied people. From a business perspective, this possibility, jointly with the commercial relevance of the CYBATHLON, could expand the market of assistive technologies from a small-scaled niche that creates little incentives for the industry to pull the technology onto the market into a broader, more mature and pervasive domain of technological innovation.

Finally, the surprising media coverage and societal attention raised by the CYBATHLON 2016 could become a critical catalyzer to raising social awareness on disability and assistive technological solutions. Several international media including the British BBC, the German Deutschlandfunk, the Swiss SRF, and the Canadian CTV provided live coverage and subsequent analysis of the competition. This degree of international coverage in mainstream media could be a ground-breaker in the effort of raising social attention and awareness about novel technological possibilities in rehabilitation. In addition, the possibility of watching real-time successful applications of current assistive technologies may contribute in changing negative societal perceptions on these products and disseminate information and knowledge about this ever evolving technological domain across society.

## Conclusion

As the fields of assistive technology and neuroengineering are entering a new phase of clinical and commercial maturity, there is an increasing need to address the ethical implications associated with the design and development of novel assistive and rehabilitative technological solutions. After reviewing various ethically-sensitive approaches to the design of NART, we proposed a framework for ethical design and development, which we call the Proactive Ethical Design (PED) framework. This framework is characterized by the convergence of user-centered and value-sensitive approaches to product design through a proactive mode of ethical evaluation. Four basic normative requirements are necessary for the realization of this framework: minimization of power imbalances, compliance with biomedical ethics, translationality and social awareness.

Cooperative efforts of researchers, end-users, clinicians and societal stakeholders are necessary to drive assistive and rehabilitation technology towards the PED framework and maximize the benefits of NART for individual users and society at large. The innovative paradigm of the CYBATHLON competition provides a promising operative model of this ethical framework and could drive an ethical shift in neuroengineering and rehabilitation. In fact, the CYBATHLON establishes a platform for exchange and cooperation among various stakeholders including people with disabilities, researchers, developers, funding actors, media and the general public. In addition, it encourages a convergence of goals between researchers and end-users, promotes compliance with ethical considerations, facilitates successful translation of new technology and raises social awareness on assistive technology and disability.

## Footnotes

1 See: http://www.resna.org/ (last accessed: 02/28/2017)

2 See: http://www.conehealth.com/app/files/public/3030/Empowering-Parkinsons-and-stroke-patients.pdf

3 See: http://www.resna.org/get-certified/code-ethics/code-ethics (last accessed: 02/19/2017).

4 See: https://ncats.nih.gov/about
